# The choice of screw internal fixation and hemiarthroplasty in the treatment of femoral neck fractures in the elderly: a meta-analysis

**DOI:** 10.1186/s13018-020-01958-2

**Published:** 2020-09-21

**Authors:** Shuai Cui, Dehui Wang, Xuejie Wang, Zehui Li, Wenlai Guo

**Affiliations:** 1 School of Mathematics, Jilin University, Changchun, Jilin Province China; 2 Department of Hand Surgery, The Second Hospital of Jilin University, Changchun, Jilin Province China

**Keywords:** Femoral neck fractures, The elderly, Internal fixation, Hemiarthroplasty, Meta-analysis

## Abstract

**Background:**

Femoral neck fractures are common fractures in the elderly. Common treatment options include internal fixation (IF) and hemiarthroplasty (HA). However, the clinical application of these two options is always controversial due to the potential clinical trauma, postoperative function, early complications, and other factors.

**Materials and methods:**

Randomized controlled trials and cohort studies comparing screw fixation and hemiarthroplasty in elderly patients with displaced femoral neck fractures were extracted from databases such as PubMed, Web of Science, EMBASE, and Cochrane. The revised Jadad scale or NOS treatment evaluation form was used to evaluate the quality of the included studies. After extracting the data, the standard deviation of continuous data and the relative risk of binary data were used. The operation time, blood loss during operation, EQ-5D (EuroQol-5 Dimension) score, mortality rate, reoperation rate, and postoperative common complications were reviewed using Review Manager software (RevMan 5.3) were compared.

**Results:**

There were 7 randomized controlled trials and 5 cohort studies. The results showed that the operation time, intraoperative blood loss, and short-term EQ-5D score of the internal fixation group were lower than those of the hemi-hip replacement group, but the reoperation rate was higher. There was no statistically significant difference in mortality and common complications such as deep vein thrombosis, pulmonary embolism, infection, and pressure sores during short-term follow-up.

**Conclusions:**

In the treatment of elderly femoral neck fractures, the screw internal fixation group has shorter operation time and less intraoperative bleeding, and the perioperative advantage is more obvious. However, the hemi-hip replacement group had more advantages in postoperative functional scoring and reoperation.

## Introduction

Femoral neck fracture is a common orthopedic injury, which is more common in the elderly. Due to the severe osteoporosis in the elderly, low energy trauma can cause femoral neck fracture, which has become one of the main threats to the decline of the quality of life or death in the elderly [1, 2]. Conservative treatment of complications and secondary mortality is high. Surgery has become the first choice of treatment for femoral neck fractures, but surgical treatment options are diversified, and there is controversy in clinical options. Surgical treatment mainly includes internal fixation and joint replacement. For young people with good bone quality, internal fixation is mostly used [3]. However, due to the high incidence of postoperative complications in elderly patients, surgical trauma often causes perioperative death and long-term dysfunction, which makes the choice of treatment options still controversial.

In order to reduce clinical heterogeneity, the most commonly used closed screw internal fixation was default plan in this study due to the advantages of less damage to soft tissue, less intraoperative blood loss, shorter operation time, and higher healing rate [4–6]. However, the requirement to stay in bed after surgery may lead to increased complications such as deep vein thrombosis, pulmonary embolism, and pressure sores. Joint replacement is only included in the more commonly used hemi-hip replacement, which is usually considered to reduce the reoperation rate and improve the postoperative functional recovery of patients [6–8], but due to the large surgical trauma, it may bring higher mortality.

In this study, after strict literature screening, the operation time, intraoperative blood loss, 1-year and 2-year EQ-5D (EuroQol-5 Dimension) scores, 1-year and 2-year mortality, reoperation rate, common postoperative complications (deep vein thrombosis, pulmonary embolism, postoperative infection and pressure sores), and other aspects of the above two treatment options were comprehensively evaluated, in order to provide evidence-based medicine for clinical selection.

## Materials and methods

This article is designed and implemented in accordance with the requirements of the PRISMA guidelines [9]. All analyses are based on previously published studies, so ethical approval and patient consent are not required.

### Literature retrieval strategy

Using Femoral Neck Fractures, Fracture Fixation, Fracture Fixation, Internal, and Hemiarthroplasty as search terms, online databases such as PubMed, Web of Science, EMBASE, and Cochrane as of April 3, 2020, were searched to compare screw internal fixation and semi-hip replacement treatment of femoral neck fractures in the elderly. References of related literature were manually searched.

### Inclusion and exclusion criteria

Inclusion criteria were as follows: (1) elderly patients with femoral neck fracture; (2) screw internal fixation and hemi-hip replacement; (3) report at least one of the following results: operation time, intraoperative blood loss, EQ-5D score for 1 and 2 years, 1-year and 2-year mortality, reoperation rate, and common postoperative complications (deep vein thrombosis, pulmonary embolism, postoperative infection and pressure sores); and (4) randomized controlled trial (RCT) or cohort study.

Exclusion criteria were as follows: (1) basic research on animal or cadaver specimens, (2) research that cannot be extracted or converted to valid data, (3) case reports and retrospective studies, (4) systematic review and meta-analysis, and (5) conference article without full-text.

### Literature screening

The two authors (Shuai Cui and Dehui Wang) independently screened according to the inclusion and exclusion criteria, in the order of deduplication, topics and abstract reading, and the full-text review. When a disagreement occurs, a third examiner (Wenlai Guo) will be included until a consensus was reached.

### Data extraction

The two reviewers (Shuai Cui and Dehui Wang) independently extracted relevant data from the included research and discussed and resolved with the third reviewer (Wenlai Guo) when a disagreement occurred until an agreement was reached. When necessary, direct communication with the original author via email was implemented to obtain the required information.

Baseline data included the following: main author name, publication time, fracture type, study type, number of patients, average age, male to female ratio, and follow-up time. Outcome indicators data include the following: operation time, intraoperative blood loss, 1-year and 2-year EQ-5D scores, 1-year and 2-year mortality, reoperation rate, and common postoperative complications (deep vein thrombosis, pulmonary embolism, postoperative infection, and pressure sores).

### Outcome indicators

Operation time and intraoperative blood loss: It is an important indicator to evaluate the degree of surgical trauma, and also the main cause of perioperative complications.

EQ-5D score: It is the main indicator to evaluate the quality of life after surgery. The higher the score, the higher the life treatment. In this study, the EQ-5D scores of 1 year and 2 years after surgery were compared.

Mortality: Death is the most serious complication for any disease. Elderly patients with femoral neck fractures often have surgical trauma and long-term bed-related deaths. In this study, the mortality rates of the two groups of patients were compared between 1 and 2 years.

Reoperation rate: Reoperations mainly include related operations caused by adverse reactions of internal fixation, repair surgery for loosening of the prosthesis, and joint replacement surgery required for femoral head necrosis after screw fixation. These are critical long-term efficacy indicators. The study only analyzed early data within 3 years after surgery.

Postoperative complications: Four aspects of complications, including deep vein thrombosis, pulmonary embolism, infection, and pressure sores, are important reasons that affect the patient’s postoperative life treatment and cause death.

### Quality assessment

The two authors evaluated the RCT using a modified Jadad scale, with a score of < 4 points, indicating low quality [10]. The cohort study used the Newcastle–Ottawa scale (NOS) for evaluation, with a score of < 5 points, indicative of low quality.

### Statistical analysis

Review Manager software (RevMan 5.3) was used for statistical analysis, and the chi-square test was used to analyze the heterogeneity of the included studies. When *I*^2^ > 50%, the random effect model is used to find the source of heterogeneity through sensitivity analysis and subgroup analysis; otherwise, the fixed effect model is used for combined analysis [11]. The dichotomous variable is represented by odds ratio (OR), and the continuous treatment effect variable is represented by mean difference (MD). The forest chart lists 95% confidence intervals and test results of hypothesis. If no less than 10 articles are included, a funnel chart is used to evaluate the publication bias. As recommended by the Cochrane Handbook for Systematic Reviews of Interventions [12], we examined publication bias using a funnel plot when the number of included studies was greater than or equal to 10.

## Results

**Fig. 1:**
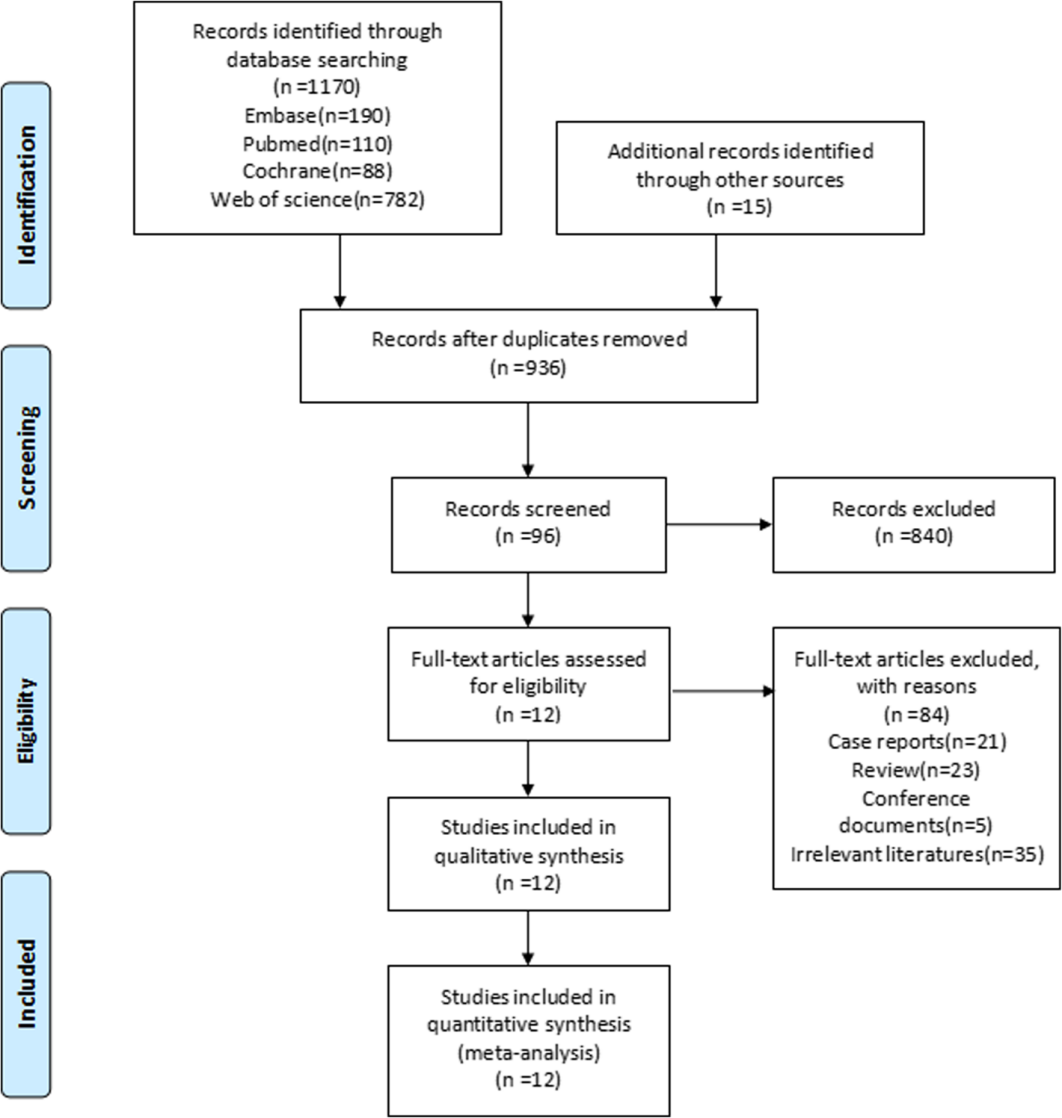
Flowchart of literature screening

As shown in the screening flowchart (Fig. 1), according to the established search strategy, we searched for PubMed, EMBASE, Cochrane, and Web of science, etc., 1170 articles in the database, and 15 articles were manually retrieved. After excluding 249 duplicate records, the remaining 96 articles were read in full text after going through the title and abstract. Among them, the article published Parker et al. in 2002 that only included the data in 2000 therefore was counted once as a 2002 article. Similarly, Gjertsen’s finding published in 2010 was not included as a 2008 study but 2010. Finally, 12 randomized controlled trials and cohort studies that met the requirements were included [13–24]. The characteristics of the included studies, baseline data, and article quality evaluation are shown in Table 1.

**Table 1:** Basic information of included studies

Research	Research period	Fracture type	Experimental design	Quality evaluation	Number of people (IF/HA)	Female (number) (IF/HA)	Average age (years) (IF/HA)	Follow-up time (months)	Outcome indicators
Puolakka et al., 2001 [13]	/	Garden III/IV	RCT	6^b^	17/15	13/14	81/82	24	Operation time, intraoperative blood loss, reoperation rate, mortality rate (2 years)
Parker et al., 2002 [14]	1991.7–2001.2	Garden III/IV	RCT	5^b^	226/229	181/183	82.2/82.4	36	Operation time, intraoperative blood loss, complications, reoperation rate, mortality rate (1 year)
Frihagen et al., 2007 [15]	2002.9–2004.3	Garden III/IV	RCT	5^b^	112/110	87/78	83.2/82.5	24	Operation time, intraoperative blood loss, complications, reoperation rate, mortality rate (1 year, 2 years), EQ-5D score (1 year, 2 years)
Hedbeck et al., 2013 [16]	2005.6–2012.5	Garden III/IV	RCT	6^b^	30/29	25/24	83.8/85.2	24	Operation time, intraoperative blood loss, complications, reoperation rate, EQ-5D score (1 year, 2 years)
Liu et al., 2016 [17]	2013.5–2013.9	Garden III/IV	RCT	5^b^	70/72	48/45	72.6/75.9	24	Reoperation rate
Lu et al., 2017 [18]	2008.1–2010.12	Garden I/II	RCT	5^b^	41/37	29/29	85.85/86.24	38.68	
Dolatowski et al., 2019 [19]	2012.2–2015.2	Garden I/II	RCT	5^b^	111/108	84/73	83.2/83.1	24	Operation time, intraoperative blood loss, complications, reoperation rate, mortality rate (1 year, 2 years), EQ-5D score (1 year, 2 years)
Partanen and Jalovaara, 2004 [20]	1989–1999	Garden III/IV	CS	4^a^	84/84	55/55	75/75	12	Reoperation rate, mortality rate (1 year)
Sikand et al., 2004 [21]	/	Garden I/II	CS	3^a^	110/29	85/21	77/79	12	Operation time, complications, reoperation rate, mortality rate (1 year)
Bjørgul and Reikerås, 2006 [22]	1998.9–2003.8	Garden III/IV	CS	6^a^	228/455	157/364	82/82	12	
Gjertsen et al., 2010 [23]	2005–2006	Garden III/IV	CS	5^a^	1823/2512	1296/1935	83.3/83.5	12	Reoperation rate, mortality rate (1 year), EQ-5D score (1 year)
Bartels et al., 2018 [24]	2005.1–2012.12	Garden III/IV	CS	6^a^	1111/1030	666/672	62.4/64.9	12	Reoperation rate, mortality rate (1 year)

*IF* internal fixation, *HA* hemi-hip replacement, *CS* cohort study, *RCT* randomized controlled study

^a^NOS score in cohort study

^b^Jadad score in RCT

### Outcome indicators

#### Operation time

**Fig. 2:**
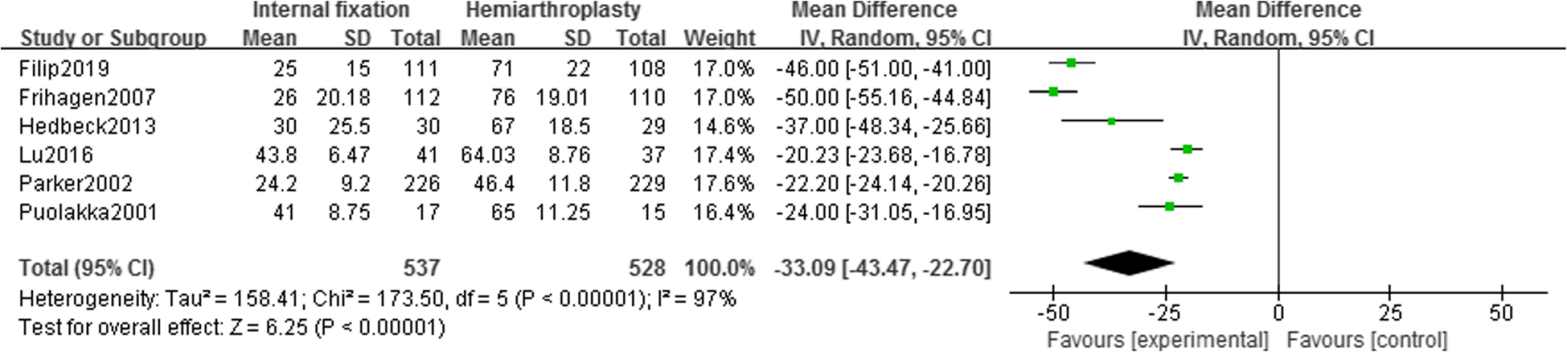
Forest map of operation time

Six studies [13–16, 18, 19] reported the operation time of 1065 patients (IF, 537; HA, 528). Using the random effect model (*I*^2^ = 97%), it was found that the operation time of the HA group was longer than that of the IF group, and there was a statistical difference [MD = − 33.09, 95% CI (− 43.47~− 22.70), *P* < .00001] (Fig. 2). No heterogeneity source was found in the sensitivity analysis.

### Intraoperative blood loss

**Fig. 3:**
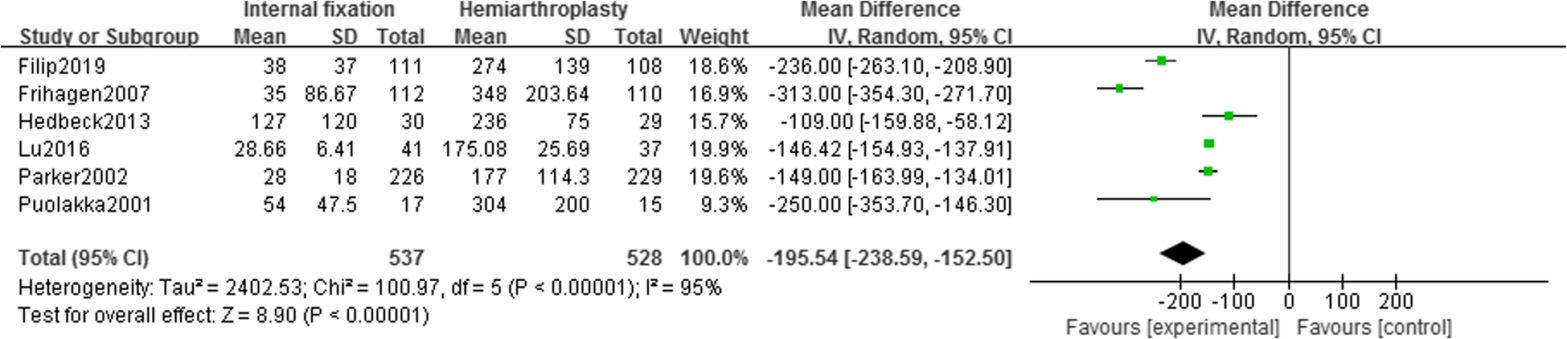
Forest map of intraoperative blood loss

Six studies [13–16, 18, 19] reported intraoperative blood loss in 1065 patients (IF, 537; HA, 528). Using the random effect model (*I*^2^ = 95%), it was found that the intraoperative blood loss in the HA group was higher than that in the IF group, and there was a statistical difference [MD = − 195.54, 95% CI (− 238.59~− 152.50), *P* < .00001] (Fig. 3). No heterogeneity source was found in the sensitivity analysis.

### EQ-5D score

#### One-year EQ-5D score

**Fig. 4:**
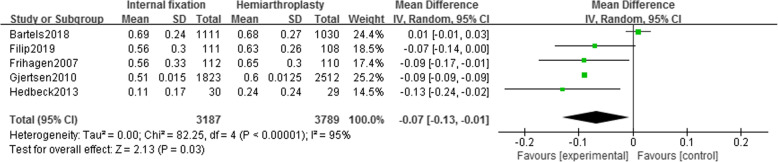
Forest map of 1-year EQ-5D score

Five studies [15, 16, 19, 23, 24] reported EQ-5D scores of 6976 patients (IF, 3187; HA, 3789) at 1-year follow-up. Random effect model (*I*^2^ = 95%) showed that the EQ-5D score of the IF group is significantly lower than that of the HA group [MD = − 0.07, 95% CI (− 0.13~− 0.01), *P* = .03] (Fig. 4). Sensitivity analysis after removing Bartels’ study revealed unchanged conclusion (*I*^2^ = 0%), i.e., the hip function recovery of the HA group was significantly better than IF group after 1 year (Supplemental Figure 1).

#### Two-year EQ-5D score

**Fig. 5:**
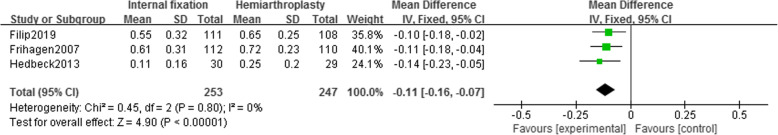
Forest map of 2-year EQ-5D score

Three studies [15, 16, 19] reported EQ-5D scores of 500 patients (IF, 253; HA, 247) at 2 years of follow-up. Using a fixed-effects model (*I*^2^ = 0%), it is shown that the EQ-5D score of the IF group is lower than that of the HA group, with statistical differences [MD = − 0.11, 95% CI (− 0.16~− 0.07), *P* < .00001], indicating that the hip function recovery of patients in HA group after 2 years was significantly better than that of IF (Fig. 5).

### Mortality

#### One-year mortality rate

**Fig. 6:**
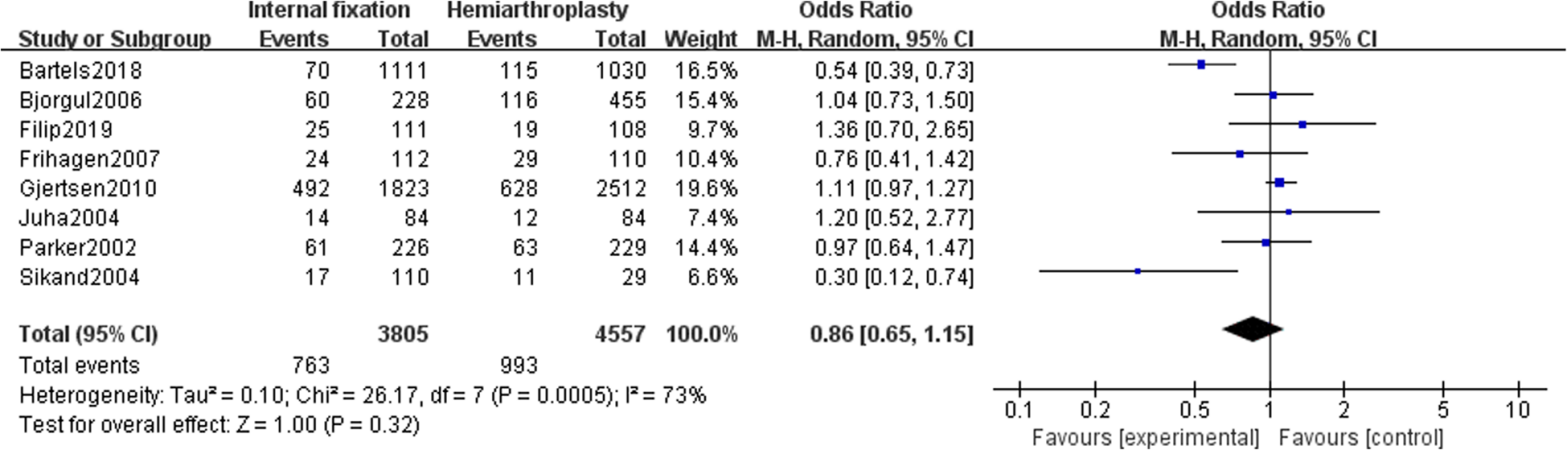
Forest map of 1-year mortality

Eight studies [14, 15, 19–24] reported a mortality rate of 8362 patients (IF, 3805; HA, 4557) followed up for 1 year. Random-effects model (*I*^2^ = 73%) suggested no significant difference in mortality between the two groups after 1 year [OR = 0.86, 95% CI (0.65~1.15), *P* = .32] (Fig. 6). After removing Bartels’ study, sensitivity analysis conclusion is unchanged (*I*^2^ = 38%) (Supplemental Figure 2).

#### Two-year mortality rate

**Fig. 7:**
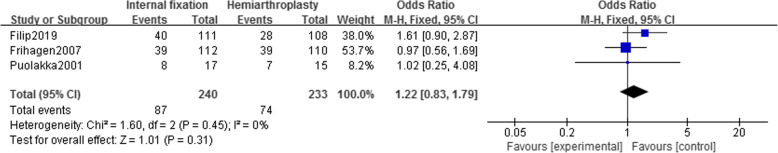
Forest map of 2-year mortality

Three studies [13, 15, 19] reported a mortality rate of 473 patients (IF, 240; HA, 233) for 2 years of follow-up. The results of the fixed-effects model (*I*^2^ = 0%) show that there is no significant difference in the 2-year postoperative mortality rate between the two groups [OR = 1.22, 95% CI (0.83~1.79), *P* = .31] (Fig. 7).

### Reoperation rate

**Fig. 8:**
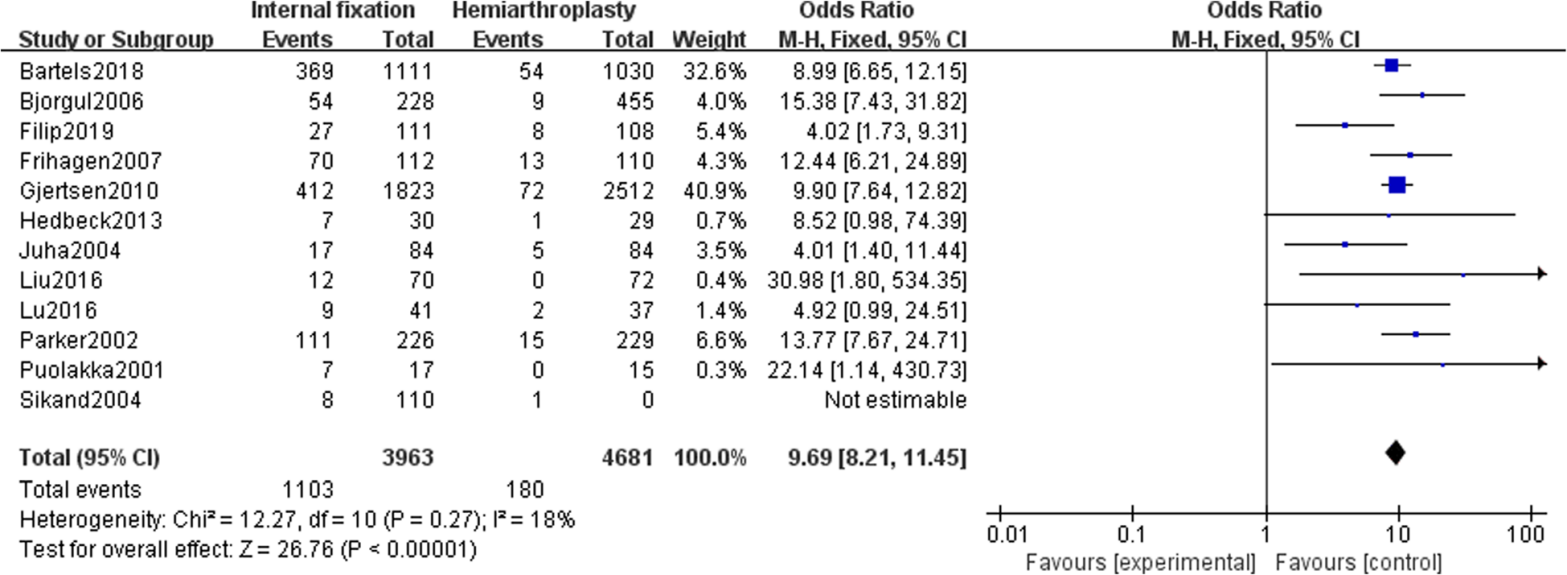
Forest chart of reoperation rate

Twelve studies [13–24] reported the reoperation rate of 8644 patients (3963 internal fixations and 4681 hemi-hip replacements). The fixed effect model (*I*^2^ = 18%) is used to summarize the data. There was a significant difference in the reoperation rate between the two groups [OR = 9.69, 95% CI (8.21~11.45), *P* < .00001]. The reoperation rate of patients with hemi-hip replacement was significantly lower than that of the internal fixation (Fig. 8).

### Complication rate

#### Incidence of deep vein thrombosis

**Fig. 9:**
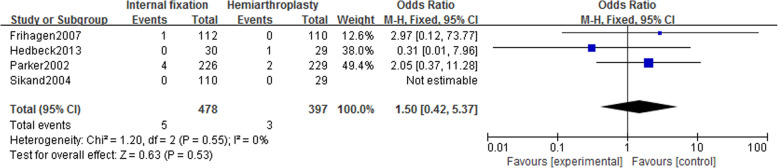
Forest map of the incidence of deep vein thrombosis

Four studies [14–16, 21] included in the study reported deep vein thrombosis in 875 patients (IF, 478; HA, 397). Using a fixed-effect model (*I*^2^ = 0%), no statistically significant difference was revealed in the incidence of deep vein thrombosis between the two groups [OR = 1.50, 95% CI (0.42~5.37), *P* = .53] (Fig. 9).

#### Incidence of pulmonary embolism

**Fig. 10:**
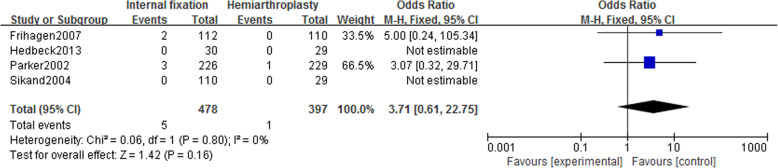
Forest map of pulmonary embolism

Four studies [14–16, 21] reported the incidence of pulmonary embolism in 875 patients (IF, 478; HA, 397). Using a fixed-effect model (*I*^2^ = 0%), there was no statistically significant difference in the incidence of pulmonary embolism between the two groups [OR = 3.71, 95% CI (0.61~22.75), *P* = .16] (Fig. 10).

#### Postoperative infection

**Fig. 11:**
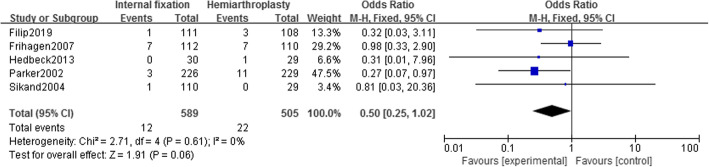
Forest map of postoperative infection

Five studies [14–16, 19, 21] reported the incidence of postoperative infection in 1094 patients (IF, 589; HA, 505). Using a fixed-effect model (*I*^2^ = 0%), there was no statistically significant difference in the incidence of postoperative infection between the two groups [OR = 0.50, 95% CI (0.25~1.02), *P* = .06] (Fig. 11).

#### Pressure sores

**Fig. 12:**
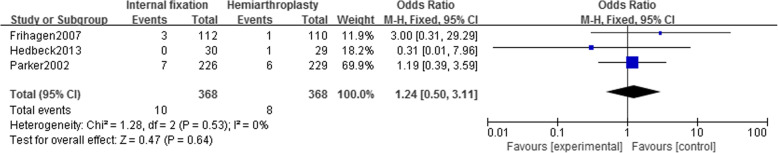
Forest map of pressure sores

Three studies [14–16] reported incidence rate of pressure sores in 736 patients (IF, 368; HA, 368). Using fixed-effect model (*I*^2^ = 0%), there was no statistical difference in the incidence rate of pressure sores in the two groups [OR = 1.24, 95% CI (0.50~3.11), *P* = .64] (Fig. 12).

## Discussion

The current study revealed that for the elderly patients with femoral neck fracture, the internal fixation group has more advantages in terms of operation time and intraoperative blood loss, but the short-term (1 year and 2 years after surgery) EQ-5D score is lower than that of hemi-hip replacement, suggesting a poor function and high reoperation rate in the internal fixation group. However, there was no statistically significant difference in mortality and severe perioperative complications such as deep vein thrombosis, pulmonary embolism, infection, and pressure sores during short-term follow-up between the two groups. In a 1-year EQ-5D and mortality study, sensitivity analysis disclosed that the heterogeneity was originated from a study by Bartels in 2018, which may be due to the fact that the average age of patients in this study was significantly younger than other groups.

Femoral neck fractures are the most common orthopedic emergency in the elderly, manifested by hip pain and inability to stand and walk, and fractures may induce ischemic necrosis of the femoral head, leading to serious dysfunction and even disability [6, 25, 26]. In the early stage, internal fixation was the most commonly used for femoral neck fractures, with advantages of shorter operation time and less blood loss during operation [27, 28], but because of the need for a longer period of bed rest post-surgery, it may cause poor postoperative function, and bed-related complications such as deep vein thrombosis, pressure sores, and even cause pulmonary embolism and increased mortality. In recent years, some surgeons have advocated such patients for hemi-hip replacement to reduce the bed rest time, thereby achieving reduced bed-related complications and better functional exercise, while avoiding more secondary operations due to femoral head necrosis [29, 30]. However, some studies believe that HA will increase the operation time and bleeding volume, bring about a higher infection rate and mortality caused by cardiovascular accidents during perioperative period [27]*.* Jiang et al. believed that although HA does not cause a higher chance of perioperative infection, it does not improve the hip function of patients either [29, 31]. Thus, in this study, it is necessary to conduct research on the abovementioned issues in those two treatments based on most recent literature.

Our research shows that HA does increase the time of surgery and the amount of bleeding during surgery. This is mainly due to the fact that screw fixation as an earlier surgical procedure has better clinician penetration and mastery. What is more, in most cases, the surgery can be closed under the guidance of the C-arm, which effectively reduces intraoperative bleeding. As an open operation, HA itself is complicated in operation, which greatly increases the patient’s operation time. Hemostasis cannot be performed through a tourniquet during operation, which leads to a significant increase in bleeding caused by open surgery and osteotomy compared with screw fixation. This conclusion is consistent with many previous studies [27, 28, 30]. The research on these two indicators (intraoperative blood loss and operation time) in this study showed a high degree of heterogeneity, likely because the data in two study are indirect data estimated according to the Cochrane transformation formula [13, 14]. Traditionally speaking, longer open surgery may bring higher postoperative infection rate, and more intraoperative blood loss will also induce higher cardiovascular and cerebrovascular accidents, leading to increased mortality, but in this study, we show that IF and HA have no difference in postoperative infection rate or mortality.

In this study, in order to evaluate the hip function early in the postoperative period, we compared the EQ-5D scores 1 year and 2 years after operation and found that HA can bring better postoperative function, which is also confirmed by Gao et al. [30]. We speculated that patients can exercise as early as possible after hemiarthroplasty to achieve functional recovery, and the bed rest time after internal fixation is relatively longer, resulting in untimely exercise and limited functional recovery. However, the functional weakness of the IF group became less obvious during long-term follow-up [29, 31], which may be due to that better exercise achieved a more satisfactory functional recovery in the later period of the IF group. The literature on the EQ-5D score in this study is relatively rare, especially that there are only 3 studies in the 2-year group, and the results are mainly based on the subjective score of the patient, without no further accurate functional evaluation. In addition, although the long-term bed rest is previously regarded to not only affect the functional recovery of patients, but also increase the incidence of deep vein thrombosis and bedsores, we did not found significant differences between two groups in occurrence of two complications.

There are eight and three studies reporting the mortality of patients 1 and 2 years after surgery, respectively, with no statistical difference found between the two groups. As mentioned earlier, although more severe surgical trauma and intraoperative blood loss are thought to lead to increased mortality in the HA group, in fact, this phenomenon did not occur, which is consistent with the results from Tseng et al. and Fisher et al. [27, 28, 30–33].

The reason for the reoperation is mainly due to necrosis of the femoral head in the IF group, or the loosening of the prosthesis in the HA group and peripheral infections. A longer follow-up time is often required for consideration of reoperation, especially the surgical reasons related to the HA group. However, the follow-up period of this study is mostly 1–2 years, with longest being only 3 years, which are ideally expected to be longer. In the current study, the reoperation rate of IF group was significantly higher than that of HA group. The reason for this phenomenon is that the femoral neck fracture in the IF group damages the blood supply of the femoral head and causes higher incidence of femoral head necrosis and nonunion. Especially, the poor bone mass and bone density are often associated with elderly patients, and once bone nonunion and osteonecrosis occur, a second joint replacement will be required.

In this study, we investigated the previously focused common serious complications such as deep vein thrombosis, pulmonary embolism, infection, and pressure sores and found no difference in the incidence of the four complications between the two groups.

## Conclusion

In the treatment of elderly femoral neck fractures, the screw IF group has shorter operation time and less intraoperative bleeding, and the perioperative advantage is more obvious, but the HA group has more postoperative functional score and less reoperation rate.

## Advantages and limitations

### Advantages

(1) Our meta-analysis established strict inclusion and exclusion criteria, while including cohort studies and randomized controlled trials to obtain a more comprehensive literature pool. (2) The internal fixation group only included screw internal fixation and excluded those fixation with a combination of pinning, nail plate, or screw plate, and only the hemi-hip joint replacement was included in the joint replacement group, all of which were expected to reduce the clinical heterogeneity.

### Limitations

(1) We did not find the source of heterogeneity in the study of the operation time and intraoperative bleeding. (2) The difference in the sample size of the included studies is relatively obvious, which may affect the stability of the conclusion. (3) In the study design, the EQ-5D score was selected as the main reference for patients to evaluate hip function after surgery, and consequently, there are few relevant data. (4) In regard to mortality, functional evaluation, and reoperation rate, there is a lack of follow-up data for more than 5 years or even longer.

## Supplementary information

**Additional file 1.** Supplemental Figure 1: Forest map of one-year EQ-5D score.

**Additional file 2.** Supplemental Figure 2: Forest map of one-year mortality.

**Additional file 3.** PRISMA 2009 checklist

## Data Availability

The two reviewers (Shuai Cui and Dehui Wang) independently extracted relevant data from the included research and discussed and resolved with the third reviewer (Wenlai Guo) when a disagreement occurred until an agreement was reached. When necessary, direct communication with the original author via email was implemented to obtain the required information.

## Abbreviations

IF: Internal fixation
HA: Hemiarthroplasty
EQ-5D: EuroQol-5 Dimension
CI: Confidence interval
ROM: Range of motion
NOS: Newcastle–Ottawa scale
RR: Relative risk
SD: Standard deviation
SMD: Standard mean difference
RCT: Randomized controlled trial
