# Supplementary material for: The choice of screw internal fixation and hemiarthroplasty in the treatment of femoral neck fractures in the elderly: a meta-analysis
Source: J Orthop Surg Res. 2020 Sep 21;15:433. doi: 10.1186/s13018-020-01958-2 (PMC7507610; doi:10.1186/s13018-020-01958-2)
Supplement: Supplementary file 2 [file 13018_2020_1958_MOESM1_ESM.docx]

	Internal fixation			Hemiarthroplasty				Mean Difference
Study or Subgroup	Mean	SD	Total	Mean	SD	Total	Weight	IV, Fixed, 95% CI
Bartels2018	0.69	0.24	1111	0.68	0.27	1030	0.0%	0.01 [-0.01, 0.03]
Filip2019	0.56	0.3	111	0.63	0.26	108	0.0%	-0.07 [-0.14, 0.00]
Frihagen2007	0.56	0.33	112	0.65	0.3	110	0.0%	-0.09 [-0.17, -0.01]
Gjertsen2010	0.51	0.015	1823	0.6	0.0125	2512	100.0%	-0.09 [-0.09, -0.09]
Hedbeck2013	0.11	0.17	30	0.24	0.24	29	0.0%	-0.13 [-0.24, -0.02]
**Total (95% CI)**			**2076**			**2759**	**100.0%**	**-0.09 [-0.09, -0.09]**

Heterogeneity: Chi² = 0.82, df = 3 (P = 0.84); I² = 0%
Test for overall effect: Z = 208.92 (P < 0.00001)

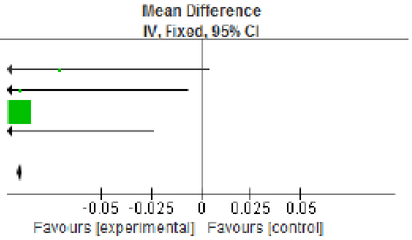

Supplemental Figure 1: Forest map of one-year EQ-5D score.
