# Supplementary material for: The choice of screw internal fixation and hemiarthroplasty in the treatment of femoral neck fractures in the elderly: a meta-analysis
Source: J Orthop Surg Res. 2020 Sep 21;15:433. doi: 10.1186/s13018-020-01958-2 (PMC7507610; doi:10.1186/s13018-020-01958-2)
Supplement: Supplementary file 3 [file 13018_2020_1958_MOESM2_ESM.docx]

	Internal fixation		Hemiarthroplasty			Odds Ratio
Study or Subgroup	Events	Total	Events	Total	Weight	M-H, Fixed, 95% CI
Bartels2018	70	1111	115	1030	0.0%	0.54 [0.39, 0.73]
Bjorgul2006	60	228	116	455	10.4%	1.04 [0.73, 1.50]
Filip2019	25	111	19	108	2.7%	1.36 [0.70, 2.65]
Frihagen2007	24	112	29	110	4.2%	0.76 [0.41, 1.42]
Gjertsen2010	492	1823	628	2512	70.0%	1.11 [0.97, 1.27]
Juha2004	14	84	12	84	1.8%	1.20 [0.52, 2.77]
Parker2002	61	226	63	229	8.3%	0.97 [0.64, 1.47]
Sikand2004	17	110	11	29	2.7%	0.30 [0.12, 0.74]
**Total (95% CI)**		**2694**		**3527**	**100.0%**	**1.06 [0.95, 1.19]**
Total events	693		878			

Heterogeneity: $Chi^2 = 9.71$, df = 6 (P = 0.14); $I^2 = 38\%$
Test for overall effect: Z = 1.04 (P = 0.30)

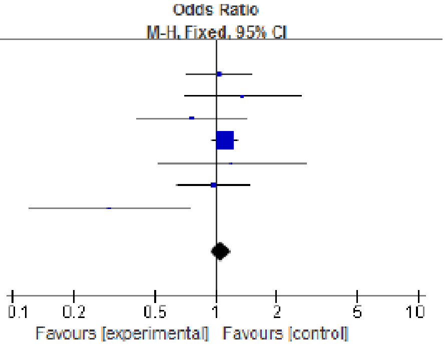

Supplemental Figure 2: Forest map of one-year mortality.
